# Noninvasive ventilation in patients with acute hypoxemic respiratory failure: a systematic review and meta-analysis of randomized controlled trials

**DOI:** 10.1038/s41598-023-35323-0

**Published:** 2023-05-22

**Authors:** Pantaree Aswanetmanee, Chok Limsuwat, Kittipong Maneechotesuwan, Phunsup Wongsurakiat

**Affiliations:** 1 Division of Respiratory Diseases and Tuberculosis, Department of Medicine, Faculty of Medicine Siriraj Hospital, Mahidol University, 2 Wanglang Road, Bangkoknoi, Bangkok, 10700 Thailand; 2 Pulmonary and Critical Care Department, Bumrungrad International Hospital, Khet Watthana, Bangkok, 10110 Thailand

**Keywords:** Diseases, Health care, Medical research

## Abstract

The clinical benefits of noninvasive ventilation (NIV) for patients with acute hypoxemic respiratory failure (AHRF) is still inconclusive. We aimed to evaluate the effect of NIV compared with conventional oxygen therapy (COT)/high-flow nasal cannula (HFNC) in this patient population. We searched for relevant studies from PubMed, Embase, Cochrane Library, ClinicalTrials.gov, CINHAL, Web of Science up to August 2019 for randomized controlled trials (RCTs) that compared NIV with COT/HFNC in AHRF. The primary outcome was the tracheal intubation rate. Secondary outcomes were intensive care unit (ICU) mortality, and hospital mortality. We applied the GRADE approach to grade the strength of the evidence. Seventeen RCTs that recruited 1738 patients were included in our meta-analysis. When comparing NIV versus COT/HFNC, the pooled risk ratio (RR) for the tracheal intubation rate was 0.68, 95% confidence interval (CI) 0.52–0.89, p = 0.005, *I*^2^ = 72.4%, low certainty of evidence. There were no significant differences in ICU mortality (pooled RR = 0.87, 95% CI 0.60–1.26), p = 0.45, *I*^2^ = 64.6%) and hospital mortality (pooled RR = 0.71, 95% CI 0.51–1.00, p = 0.05, *I*^2^ = 27.4%). Subgroup analysis revealed that NIV application with helmet was significantly associated with a lower intubation rate than NIV with face mask. NIV did not show a significant reduction in intubation rate compared to HFNC. In conclusion, NIV application in patients with medical illness and AHRF was associated with a lower risk of tracheal intubation compared to COT. NIV with helmet and HFNC are promising strategies to avoid tracheal intubation in this patient population and warrant further studies. NIV application had no effect on mortality.

The study protocol was registered in the International Prospective Register of Systematic Reviews (PROSPERO; CRD42018087342).

## Introduction

Noninvasive ventilation (NIV) refers to the delivery of mechanical ventilation with techniques that do not require an invasive endotracheal airway^1^. NIV has been increasingly used worldwide. Currently, it is the first-line preferred therapy for acute on chronic ventilatory failure, mainly for exacerbations of chronic obstructive pulmonary disease (COPD). However, the use of NIV in the treatment of acute hypoxemic respiratory failure (AHRF) remains controversial. AHRF is a major problem in acute care settings in adult patients, often leading to endotracheal intubation and invasive mechanical ventilation. The hallmark of AHRF is severe hypoxemia (PaO_2_/FiO_2_ ≤ 300 mmHg) that requires high levels of oxygen and is accompanied by clinical signs of respiratory distress. The clinical evidence supporting the use of NIV in this condition have yielded conflicting results. NIV may improve oxygenation, facilitate ventilation, reduce work of breathing and dyspnea, avoid intubation, and reduce complications associated with invasive mechanical ventilation^2^. An important concern of using NIV for the indication of AHRF is the delay in endotracheal intubation that leads to higher mortality in patients with NIV failure^3^. Furthermore, concerns about the safety of NIV for patients with AHRF have recently been raised based on possible associations with higher tidal volumes leading to perpetuating lung injury in patients with high respiratory effort and increased mortality in some studies^4–6^. Recently, high-flow nasal cannula (HFNC) therapy has been shown to offer several advantages compared with NIV, including easier application and better patient tolerance^7,8^. Despite all these concerns, recent epidemiological data show that NIV is routinely used in patients with hypoxemic ARF and can be applied as first-line ventilatory support in 15–30% of them^9–11^.

Because of the controversy regarding the effectiveness of NIV in patients with AHRF, the safety concern of NIV, and the arrival of HFNC, we conducted a systematic review and meta-analysis of published randomized controlled trials (RCTs) to examine the effects of NIV on the tracheal intubation rate and mortality compared to conventional oxygen therapy (COT: oxygen cannula, oxygen face mask, or venturi face mask) and/or HFNC in adult patients with AHRF.

## Methods

This review was carried out according to the Preferred Reporting Items for Systematic Review and Meta-analysis Protocols (PRISMA-P) and the Patient, Intervention, Comparison, and Outcome (PICO) model was used. The protocol was registered in the International Prospective Register of Systematic Reviews (PROSPERO; CRD42018087342). Institutional review board approval was not required because all study data had been previously published and this study did not include individual patient data.

### Search strategy

#### Information sources

We searched PubMed, Embase, the Cochrane Library, ClinicalTrials.gov, CINAHL, and Web of Science using the following keywords (“noninvasive ventilation” OR “NIV” OR “noninvasive positive pressure ventilation” OR “NIPPV” OR “continuous positive airway pressure” OR “CPAP” or “bilevel positive airway pressure” OR “BiPAP”) AND (“acute lung injury” OR “ALI” OR “acute respiratory distress syndrome” OR “ARDS” OR “acute hypoxemic respiratory failure” OR “AHRF”. Studies from January 1995 to August 2019 were retrieved. In addition, references from retrieved papers were checked for additional studies. Data from the full published English paper were collected.

### Eligibility criteria

Inclusion criteria were (1) trials conducted in adult age ≥ 18 years, with AHRF; (2) studies comparing NIV with COT and/or HFNC; (3) the outcomes included tracheal intubation rate, ICU mortality, or hospital mortality; (4) full text of the RCTs was available and published in English language; (5) for the results of a study published more than once, only those with the most complete and up-to-date information will be included in the analysis, and; (6) for RCTs with a mixed population of hypercapnic and nonhypercapnic patients, only data from the nonhypercapnic group were retrieved.

Exclusion criteria were (1) participants were children or adolescents (< 18 years); (2) majority of the participants were COPD or postoperative state, traumatic patients or postextubation state. We exclude these participants because the pathophysiology and indication and outcomes for NIV are different from patients with AHRF; (3) patients with hypercapnia (PaCO_2_ > 50 mm Hg); (4) the trial did not use COT or HFNC as a control; (5) palliative setting; (6) the study did not include extractable outcomes or mortality data.

### Study selection criteria and procedures

To assess eligibity, two authors (P.A. and C.L.) independently reviewed the titles and abstracts generated by the literature search using the keyword terms specified above. Eligible studies were retrieved using the stated inclusion and exclusion criteria. Any discrepancies between the two authors were discussed among all four authors. The selection results from the duplicate review of articles were compared to ensure that all relevant articles were retrieved. The most complete and up-to-date information was included in the meta-analysis.

### Data abstraction

A data collection form in Excel was used to gather all the information we need from selected studies. The form was discussed and approved by all investigators. Data were extracted independently in duplicate. The results of duplicate data collection were compared. Discrepancies in data collection between the two authors were resolved by discussion and consensus. The following data on the characteristics of the trial and its participants were collected: general information about the study (e.g. title, name of the trial, authors, year of publication), study characteristics (e.g. sample size, study design, randomization, blinding, duration of follow-up, loss of follow-up), intervention information (e.g. type of interface, duration and frequency of treatment for the intervention group and control group), participant demographics (e.g. age, gender, race/ethnicity, disease severity), study outcomes (tracheal intubation rate, ICU mortality, and hospital mortality), comorbid conditions, respiratory parameters, reasons for NIV failure, complications, and method of statistical adjustment. For categorical outcomes, reviewers extracted the number of intubations, ICU death, and hospital death, along with all-cause mortality, corresponding risk ratio, confidence intervals, and P values.

### Assessment of study quality

Elements of the Cochrane Collaboration tool to assess risk of bias and PRISMA guidelines were used to examine the quality of the selected studies^12^. The elements used in this meta-analysis included random sequence generation and allocation concealment (selection bias), blinding of participants, personnel and outcome assessment (performance and detection bias), incomplete outcome data (attrition bias), and selective reporting (reporting bias). If one or more individual domains are assessed as having a high risk of bias, the trial is rated as having a high risk of bias. All domains must have been rated as having a low risk of bias for the overall risk of bias to be classified as low. In cases of unclear risk of bias or mixed assessments of low and unclear risk of bias, the overall score was classified as having an unclear risk of bias. Two authors (P.A. and C.L.) independently assessed the articles and the overall risk of bias was classified and disagreements were resolved by discussion and consensus with all four authors.

### The principal summary measures

We examined the relationship between the use of NIV therapy in AHRF patients and the outcomes by first calculating the net effect size. Intubation rate, ICU mortality, and hospital mortality are considered as dichotomous data. We derived the pooled risk ratio (RR) for dichotomous outcomes with 95% confidence intervals (CIs). The statistical significance of the Alpha level was set at 0.05.

### Data analysis

Individual RR and 95% CI for intubation rate, ICU mortality, and hospital mortality were included in the meta-analysis. Data for analysis were extracted directly and calculated from the text of each eligible article. As the studies may differ from each other in various ways and the goal of this meta-analysis is to extrapolate the result from this study population to others, the random effects model was used in the analysis. The DerSimonian and Laird random effects model was applied to calculate the pooled RR^13^. We evaluated the heterogeneity of effect size across studies with the Q statistic^14^, considering any P-value < 0.1 as evidence of heterogeneity. An *I*^*2*^ value of 25% to 49% is considered a low level of heterogeneity, 50% to 74% a moderate level, and 75% to 100% a high level^15,16^. Forest plots were constructed to demonstrate the RR and 95% CI for individual studies and the summary effect sizes. Statistical significance for z test was set at the P-value < 0.05. Heterogeneities were further explored with subgroup analysis, and sensitivity analysis. We aimed to further conduct subgroup analysis by immune status (nonimmunocompromised/immunocompromised), interface type (face mask/helmet), and type of oxygen support (COT/HFNC). Sensitivity analysis was performed to assess the robustness of our result using a leave-one-out method. Publication bias was assessed using the visual inspection of funnel plots, and Egger’s weight linear regression^17^. Statistical analyses were performed using STATA software version 14.0 (Stata Corp LP, College Station, TX).

We applied the Grading of Recommendations Assessment, Development and Evaluation (GRADE) approach in order to grade the strength of the evidence for our primary outcome (tracheal intubation rate). The assessment domains include risk of bias, inconsistency, imprecision, indirectness, and other factors. The strength of evidence is classified as high, moderate, low, or very low^18^. All authors individually evaluated the strength of the body of evidence with discrepancies resolved through consensus.

## Results

### Baseline characteristics of the included studies

**Figure 1:**
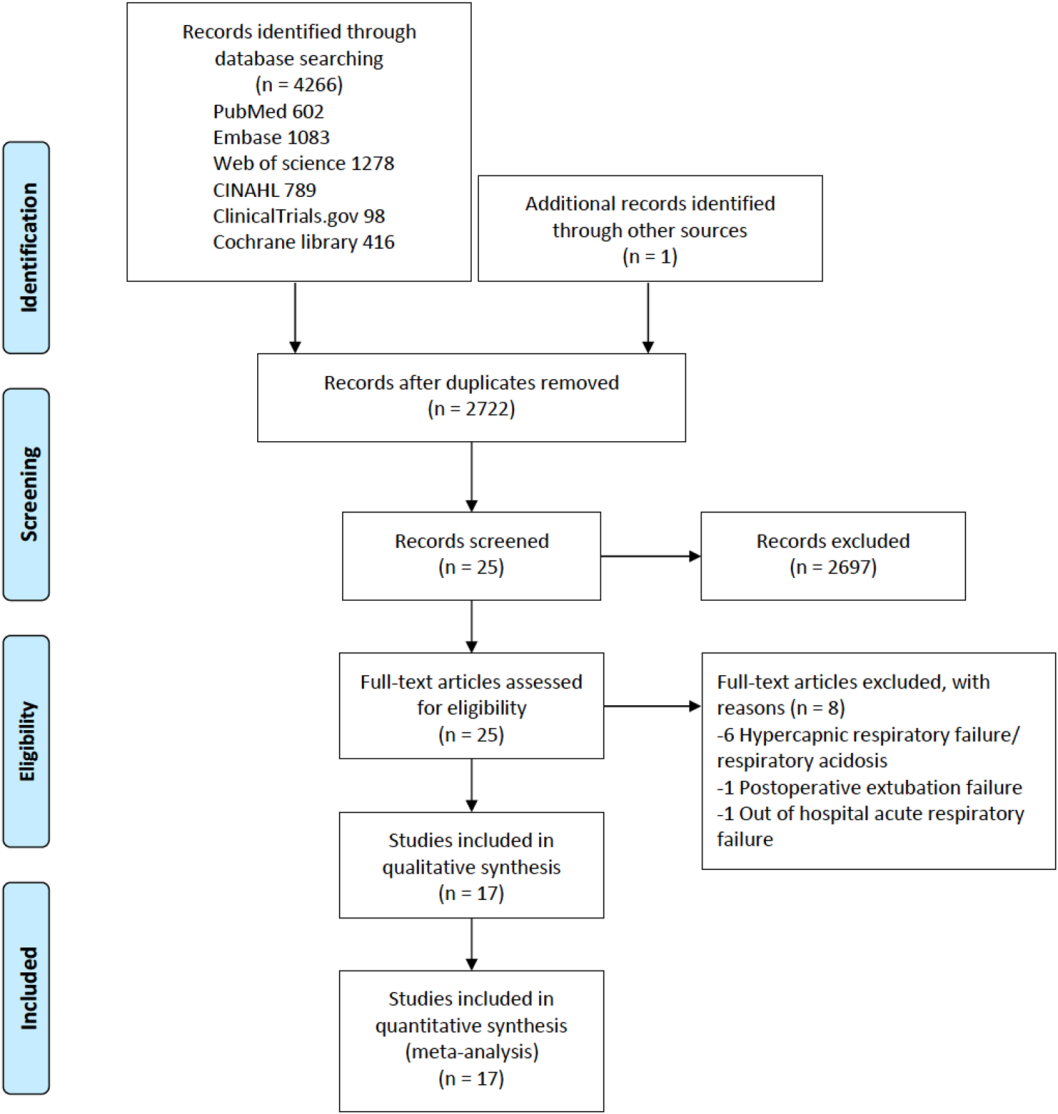
PRISMA flow diagram of trial selection.

We initially identified 4266 articles. After the duplicate articles were removed, 2722 articles were screened for eligibility criteria. A total of 17 articles^8,19–34^ were included for our systematic review and meta-analysis (Fig. 1).

Most studies compared NIV BiPAP with COT. Two studies use HFNC as control in their studies^8,32^. The primary outcome in most studies was the intubation rate. Only one study used intubation criteria as a primary outcome. One study used physiologic changes in PaO_2_/FiO_2_ as the primary outcome. One study^34^ had no intubation event or mortality in both groups, therefore it was not included in the forest plot. The baseline characteristics of the included studies are shown in Table1.

**Table 1:** Main characteristics of included studies.

Source	No. of patients	Main risk factor for AHRF	Intervention	Comparator	Outcomes of interest	
					Primary	Secondary
Wysocki (1995)	24	Mixed acute respiratory failure (pneumonia, pulmonary edema)	Face mask NIV, BiPAP	COT	Intubation rate	ICU mortality, hospital mortality, length of ICU stay
Confalonieri (1999)	33	Severe CAP	Face mask NIV, BiPAP	COT	Intubation rate	Hospital mortality, length of hospital stay, duration of MV
Antonelli (2000)	40	Solid organ transplant	Face mask NIV, CPAP	COT	Intubation rate	ICU mortality, hospital mortality, duration of MV, length of hospital stay
Delclaux (2000)	123	Acute lung injury	Face mask NIV, CPAP	COT	Intubation rate	ICU mortality, hospital mortality
Hilbert (2001)	52	Immunosuppressed patients	Face mask NIV, BiPAP	COT	Intubation rate	ICU mortality, hospital mortality, duration of MV, length of hospital stay
Ferrer (2003)	105	Mixed acute respiratory failure (pneumonia, pulmonary edema, ARDS)	Face mask NIV, BiPAP	COT	Intubation rate	ICU mortality
Nava (2003)	66	Cardiogenic pulmonary edema	Face mask NIV, BiPAP	COT	Intubation rate	Hospital mortality, dyspnea, arterial blood gas, blood pressure, heart rate
Park (2004)	80	Cardiogenic pulmonary edema	Face mask NIV, BiPAP	COT	Intubation rate	hospital mortality
Squadrone (2010)	40	Hematologic malignancy and acute lung injury	Helmet NIV, CPAP	COT	Intubation rate	Hospital mortality, length of ICU stay
Cosentini (2010)	47	CAP	Helmet NIV, CPAP	COT	PaO_2_/FiO_2_	Intubation rate
Wermke (2011)	86	Allogenic stem cell transplants patients with respiratory failure	Face mask NIV, BiPAP	COT	100-day mortality	Overall survival, intubation rate
Ducros (2011)	207	Acute pulmonary edema	Face mask NIV, CPAP	COT	Composite of death, intubation criteria, circulatory failure	Composite endpoint without intubation criteria
Zhan (2012)	40	Acute lung injury	Face mask NIV, BiPAP	COT	Intubation rate	ICU mortality, hospital mortality
Brambilla (2014)	81	CAP	Helmet NIV, CPAP	COT	Intubation rate	Hospital mortality, length of hospital stay
Lemiale (2015)	374	Immunocompromised patients with AHRF	Face mask NIV, CPAP	COT	28-day mortality	Intubation rate
Frat (2015)	310	Mixed AHRF (pneumonia)	Face mask NIV, BiPAP	COT/HFNC	Intubation rate	ICU mortality, 90-day mortality, length of ICU stay
Azevedo (2015)	30	Mixed AHRF (pneumonia, pulmonary edema)	Face mask NIV, BiPAP	HFNC	Intubation rate	–

*AHRF* acute hypoxemic respiratory failure, *MV* mechanical ventilation, *NIV* noninvasive ventilation, *CAP* community-acquired pneumonia, *BiPAP* bilevel positive airway pressure, *CPAP* continuous positive airway pressure, *COT* conventional oxygen therapy (oxygen cannula, oxygen face mask, or venturi face mask), *HFNC* high-flow nasal cannula.

### Quality assessment

**Figure 2:**
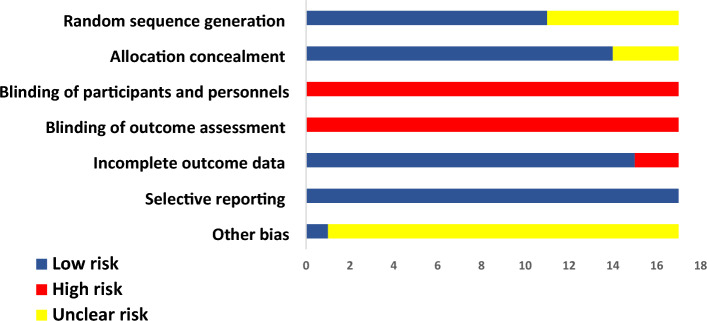
Risk of bias summary for included studies. Red indicates high risk of bias; yellow indicates unclear risk of bias; and blue indicates low risk of bias.

The risk of biases in the overall studies are shown in Fig. 2. Most studies showed a low risk of random sequence generation and allocation concealment biases. However, the nature of the intervention (NIV) could not be blinded to patients or health care personnel. Therefore, there was a considerable risk of detection bias.

### Primary outcome: tracheal intubation rate

**Figure 3:**
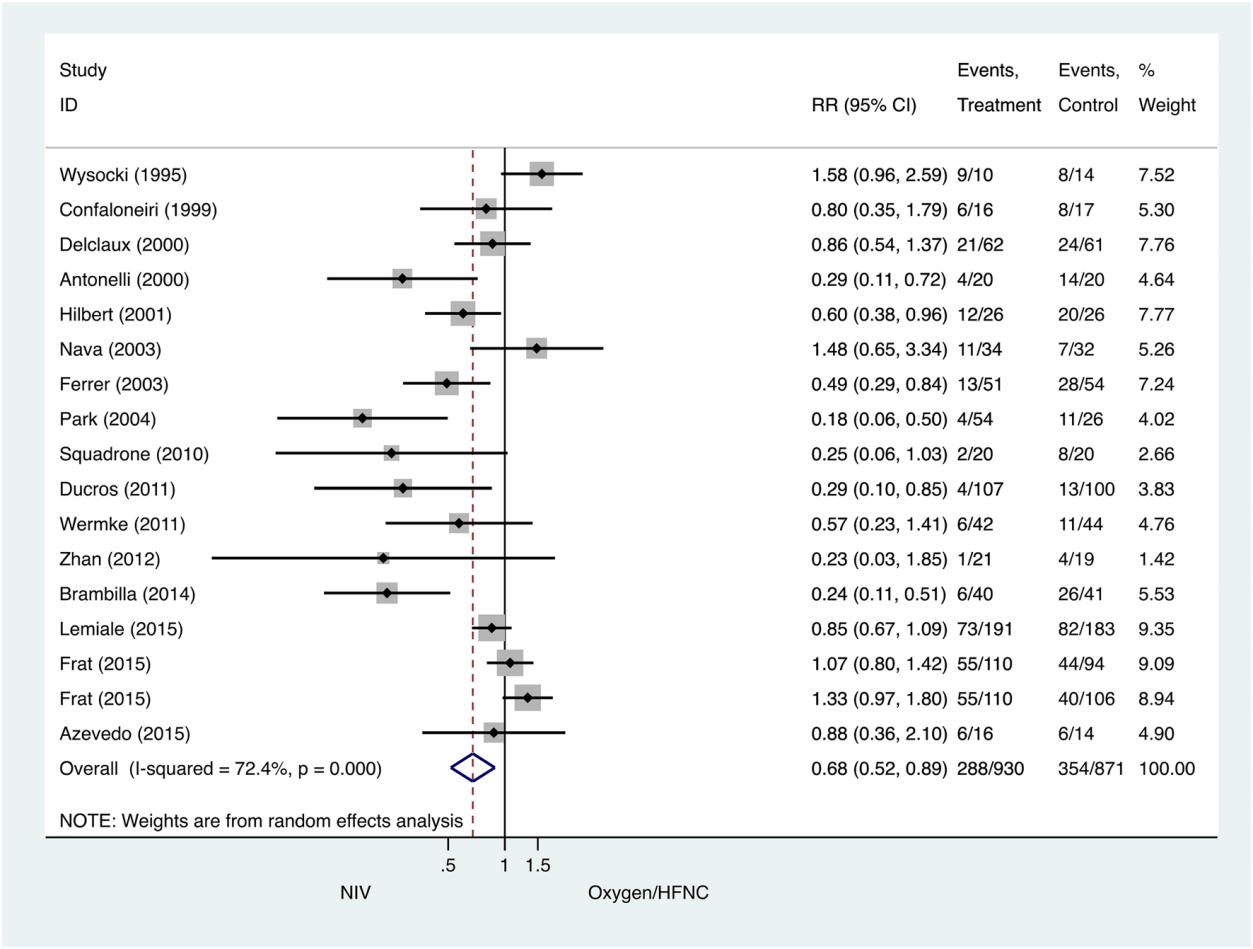
Intubation rate: noninvasive ventilation (NIV) versus conventional oxygen therapy/high-flow nasal cannula (HFNC). Boxes and horizontal lines represent point estimates and 95% confidence intervals, varying in size according to the weight in the analysis.

Seventeen trials studied the differences between the intubation rate of the NIV and COT/HFNC groups. The intubation rate was lower in the NIV group compared to the COT/HFNC group (pooled RR 0.68, 95% CI 0.52–0.89, P = 0.005) (Fig. 3).

### Secondary outcome: ICU mortality and hospital mortality

**Figure 4:**
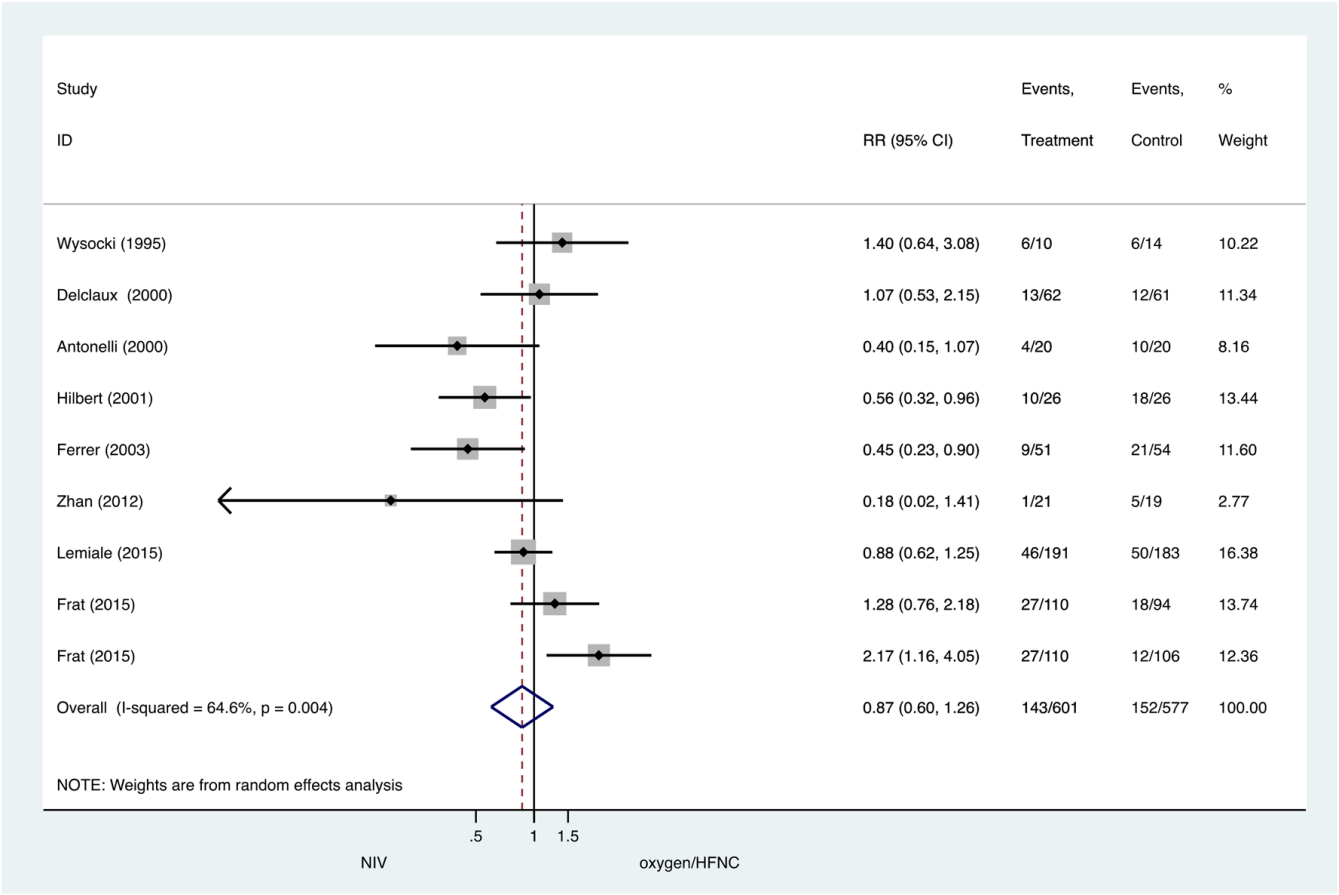
ICU mortality: noninvasive ventilation (NIV) versus conventional oxygen therapy/high-flow nasal cannula (HFNC). Boxes and horizontal lines represent point estimates and 95% confidence intervals, varying in size according to the weight in the analysis.

**Figure 5:**
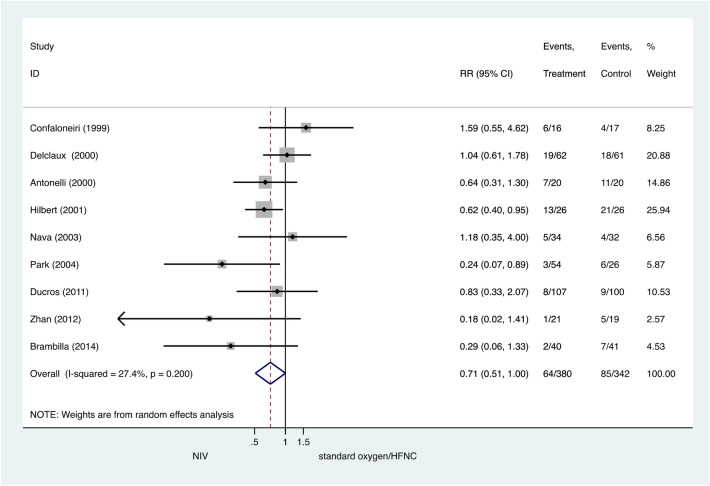
Hospital mortality: noninvasive ventilation (NIV) versus conventional oxygen therapy/high-flow nasal cannula (HFNC). Boxes and horizontal lines represent point estimates and 95% confidence intervals, varying in size according to the weight in the analysis.

There were no significant differences between the NIV and COT/HFNC groups for ICU mortality (pooled RR 0.87, 95% CI 0.60–1.26, P = 0.45) (Fig. 4) or hospital mortality (pooled RR 0.71, 95% CI 0.51–1.00, P = 0.05) (Fig. 5).

### Subgroup analysis

**Figure 6:**
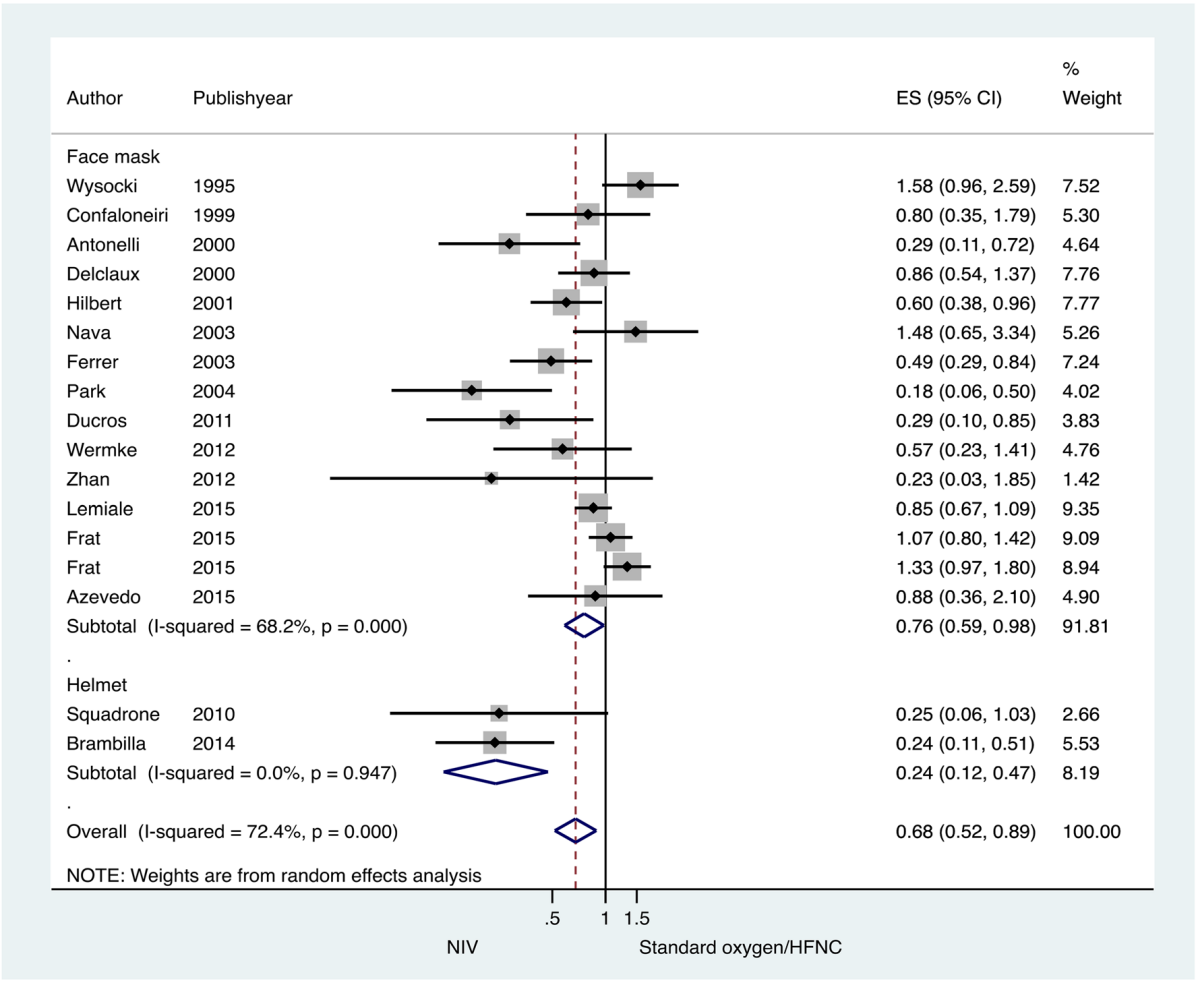
Subgroup analysis according to interface type (facemask NIV or helmet NIV): intubation rate in acute hypoxemic respiratory failure patients randomized to noninvasive ventilation (NIV) versus conventional oxygen therapy/high-flow nasal cannula (HFNC). Boxes and horizontal lines represent point estimates and 95% confidence intervals, varying in size according to the weight in the analysis.

**Figure 7:**
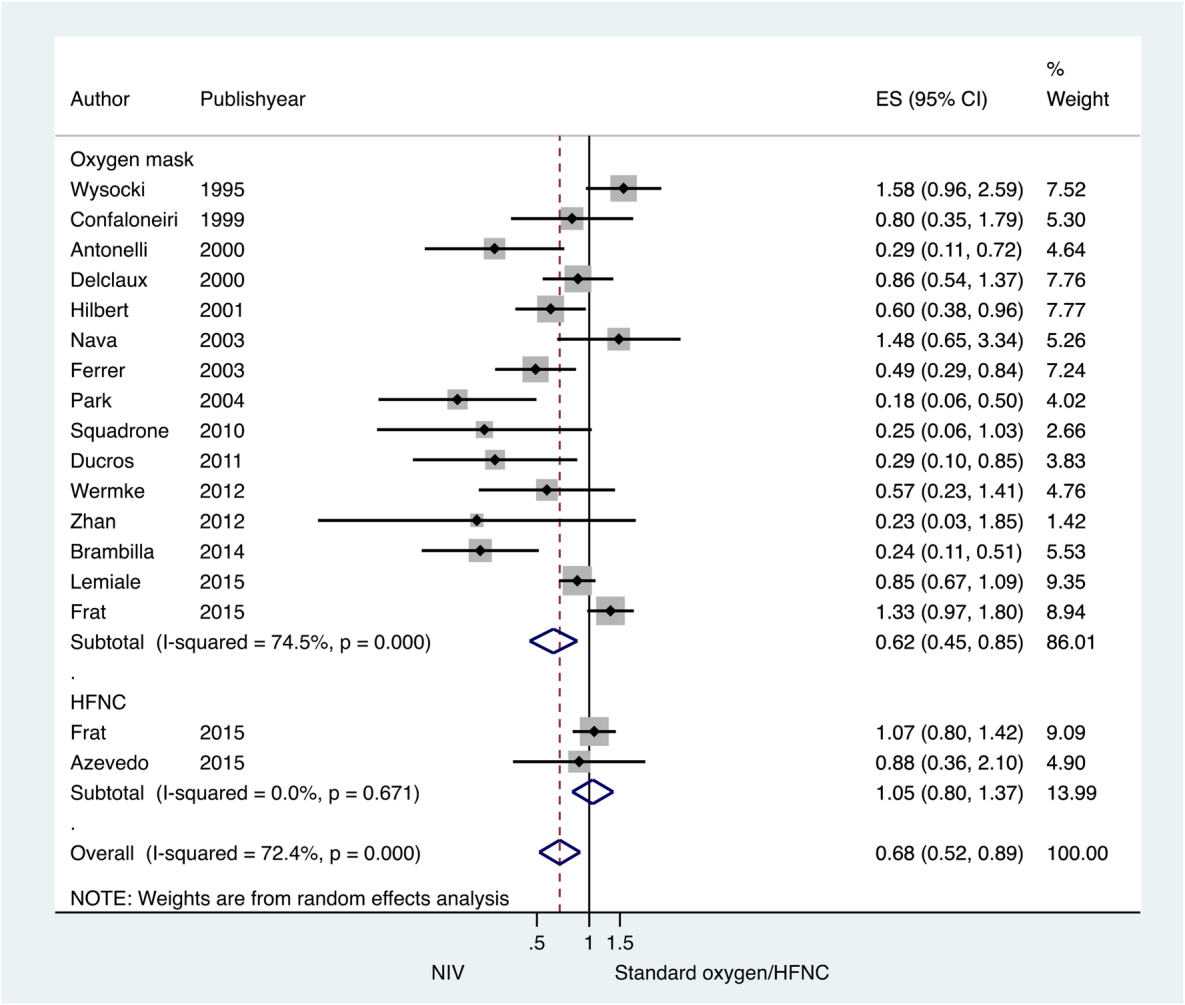
Subgroup analysis according to type of oxygen therapy [conventional oxygen therapy (COT) or high-flow nasal cannula (HFNC)]: intubation rate in acute hypoxemic respiratory failure patients randomized to NIV versus COT/HFNC. Boxes and horizontal lines represent point estimates and 95% confidence intervals, varying in size according to the weight in the analysis.

Subgroup analyses were performed to evaluate the causes of heterogeneity. The type of interface significantly caused heterogeneity in the tracheal intubation rate. The NIV application with helmet significantly reduced the intubation rate compared to the full face mask (test of heterogeneity between subgroup Q = 13.85, df = 1, P < 0.001) (Fig. 6). Subgroup analysis by immune status revealed that NIV used in immunocompromised patients led to a significant reduction in the intubation rate (test of heterogeneity between subgroup Q = 3.9, df = 1, P = 0.04) (Fig. S1). There were only two studies in the HFNC subgroup. Compared to HFNC, NIV did not result in a reduction in the intubation rate (Fig. 7).

### Sensitivity analysis

Sensitivity analysis revealed that after removing three RCTs that included some population of hypercapnic respiratory failure^21,22,26^, the primary outcome remained in the same direction (Fig. S2). NIV reduced the tracheal intubation rate compared to COT/HFNC therapy.

### Publication bias

Based on visual inspection of the funnel plot (Fig. S3), there may be some asymmetry, and the Egger linear regression test suggested the existence of publication biases (P = 0.032) in the tracheal intubation rate.

We graded the overall strength of the evidence for the primary outcome (tracheal intubation rate) as low (Table S1).

## Discussion

In our systematic review and meta-analysis of 17 randomized trials, including 1738 patients with medical illness and AHRF, NIV therapy significantly reduced the intubation rate compared to COT/HFNC. This result is consistent with previous meta-analyses^35–40^. Of the six previous reviews, three included patients with AHRF of various etiologies, one included only patients with pneumonia, and two included only immunocompromised patients. Our review focused on studies in patients with AHRF, defined as significant hypoxemia with clinical respiratory distress. We excluded patients with hypercapnic respiratory failure, mainly patients with COPD as the pathophysiology and outcome are very different from AHRF. Furthermore, the beneficial effects of NIV to reduce intubation and mortality in patients with COPD are well established. Similarly, we also excluded postoperative patients, traumatic patients and post-extubated patients. Therefore, our analysis included only patients with medical illness with AHRF for whom the role of NIV is most controversial. Patients in the studies included in our analysis predominantly presented with pneumonia, acute respiratory distress syndrome (ARDS), pulmonary edema, and AHRF in immunocompromised patients’.

We did not exclude patients with cardiogenic pulmonary edema as the etiology of AHRF is often unclear at presentation and the decision to start NIV is practically based on clinical respiratory distress together with significant hypoxemia and usually before the definitive diagnosis is known. In addition, cardiogenic pulmonary edema and ARDS may sometimes coexist^41^. Therefore, with respect to the application of NIV, we consider studies on these patients to be in the same category. This patient population poses a significant challenge within adult acute care settings, often leading to endotracheal intubation and invasive mechanical ventilation. From our analysis, compairing to COT/HFNC, NIV application may reduce the intubation rate in these patients. However, we graded the overall strength of the evidence for a reduction in the tracheal intubation rate as low since all included trials were unblinded due to the nature of the intervention (NIV), and therefore, all were considered to be at high risk of bias. In addition, there was moderate to high heterogeneity in this outcome analysis. Subgroup analysis to identify the source of heterogeneity revealed that NIV with helmet significantly reduced the intubation rate compared to full face mask. The helmet can effectively deliver a higher level of PEEP and the helmet neck seal allows a higher level of airway pressure delivery. This may correspond to a reduction in intubation rate in the helmet group^6^. Because of this significant difference in the outcome, the future studies regarding NIV should specifically define the type of interface used in the studies.

Among the 17 studies included in this analysis, nine studies reported ICU mortality and nine studies reported hospital mortality. Our analysis revealed that there were no significant difference between NIV and COT/HFNC for both ICU mortality and hospital mortality. Previous meta-analyses have yielded conflicting conclusions on the impact of NIV on mortality in patients with AHRF. Xu et al. showed that NIV significantly reduced hospital mortality^35^. This meta-analysis retrieved RCTs from inception to 2016 including six studies with 503 patients with AHRF excluding COPD and cardiogenic pulmonary edema. However, this analysis included some studies with nonmedical illness such as postoperative hypoxemia. Huang et al. analyzed immunocompromised patients with ARF, including five studies with 592 patients and reported that early use of NIV could reduce short-term mortality compared to COT^36^. In contrast, the analysis of Zayed et al. including seven studies with 664 immunocompromised patients, revealed that there were no significant differences between NIV and COT or NIV and HFNC regarding short-term mortality^37^. Ruzsics et al. analyzed patients with pneumonia-associated respiratory failure, including five studies with 121 patients and revealed that, compared to COT or invasive ventilation, NIV did not significantly reduce hospital mortality, especially if patients with COPD were excluded from the analysis^38^. Sakuraya et al. performed a network meta-analysis comparing the efficacy of noninvasive ventilation according to ventilation modes with HFNC, standard oxygen therapy (SOT), and IMV in adult patients with AHRF, excluding cardiopulmonary edema, acute exacerbation of COPD, hypercapnia, post-extubation, post-surgical status, trauma. Using SOT as a reference, CPAP was significantly associated with a lower risk of mortality. PSV and HFNC were not associated with a significantly lower risk of mortality^39^. Ferreyro et al. reported a network meta-analysis in which the efficacy of noninvasive respiratory management strategies was compared with that of SOT among adult patients with AHRF and found that both helmet NIV and facemask NIV were associated with a lower risk of mortality and intubation compared to SOT^40^. However, when excluding trials that included patients with COPD and/or congestive heart failure, face mask NIV was no longer associated with decreased mortality.

Put together, the effect of NIV on avoiding intubation in patients with AHRF seems to persist in all meta-analysis. However, the effect of NIV on mortality was inconsistent among different analyses. This may be explained by the difference between the patients included in the analyses. AHRF is a consequence of various lung diseases. Although early application of NIV may avoid intubation, the effect on mortality would depend on the balance between the beneficial effect of reducing complications associated with invasive mechanical ventilation, the reversibility and effectiveness of treatment for underlying causes of AHRF, and the well-recognized harmful effect of NIV, that is delayed endotracheal intubation. Furthermore, concerns have recently been raised about the safety of NIV for patients with AHRF based on possible associations with higher tidal volumes leading to perpetuating lung injury in patients with high respiratory effort and increased mortality in some studies^4–6^. Therefore, the effect of NIV on mortality is unpredictable and inconsistent between different studies.

Until recently, almost all studies on NIV compared it with oxygen delivered by COT. Recently, HFNC has been shown to offer several advantages compared to NIV, including promoting secretion drainage, reducing dead space and better tolerance^7^. Furthermore, in our subgroup analysis, NIV did not significantly reduce the intubation rate compared to HFNC. The use of HFNC might be diluting the benefits of NIV on the intubation rate in this analysis. However, there were only two studies evaluating HFNC in the subgroup analysis. Therefore, uncertainty remains about the effectiveness of this treatment. Further studies are required on the role of HFNC in AHRF compared to NIV.

The strength of the present study is the comprehensive systematic literature search from many databases according to the PRISMA guideline. Furthermore, we included only RCT to minimize internal biases of confounders as they usually occur in observational studies and the appraisal of internal validity was performed using the Cochrane Collaboration tool for assessing risk of bias and the PRISMA guidelines. Our analysis focuses only on patients with medical illness and AHRF and targets clinically important outcomes, including the intubation rate and mortality.

This study has some limitations. First, language restrictions may have contributed to incomplete inclusion of relevant studies. Second, the variation in baseline characteristics, severity of hypoxemia, severity of acute illness of the study population, and intervention protocols between trials may lead to a significant level of heterogeneity with insufficient data to explore relevant subgroup effects. Third, the nature of the intervention could not be blinded and may lead to different thresholds to provide endotracheal intubation, which was the primary outcome of this analysis. Fourth, because of the COVID-19 pandemic, our review and analysis was stopped in August 2019. Some studies regarding the effect of noninvasive respiratory support in patients with COVID-19 were not included in this analysis.

## Conclusions

In patients with medical illness and AHRF, NIV application was associated with a lower risk of tracheal intubation compared to COT. NIV with helmet and HFNC are promising strategies to avoid tracheal intubation in this patient population and warrant further studies. NIV application had no effect on mortality.

## Supplementary Information

Supplementary Information.

## Data Availability

The data and material used for this meta-analysis were obtained from the articles in our list of references. The datasets used in this study are available from the corresponding author upon reasonable request.

## Abbreviations

AHRF: Acute hypoxemic respiratory failure
CAP: Community-acquired pneumonia
COT: Conventional oxygen therapy
CI: Confidence interval
COPD: Chronic obstructive pulmonary disease
FiO_2_: Fraction of inspired oxygen
GRADE: Grading of Recommendations Assessment, Development and Evaluation
HFNC: High-flow nasal cannula
NIV: Noninvasive ventilation
PICO: Patient, Intervention, Comparison, and Outcome
PRISMA: Preferred Reporting Items for Systematic Review and Meta-analysis
RCT: Randomized controlled trial
RR: Risk ratio
SOT: Standard oxygen therapy
