# Supplementary material for: Noninvasive ventilation in patients with acute hypoxemic respiratory failure: a systematic review and meta-analysis of randomized controlled trials
Source: Sci Rep. 2023 May 22;13:8283. doi: 10.1038/s41598-023-35323-0 (PMC10202054; doi:10.1038/s41598-023-35323-0)
Supplement: Supplementary file 1 — Supplementary Information. [file 41598_2023_35323_MOESM1_ESM.docx]

**Noninvasive ventilation in patients with acute hypoxemic respiratory failure: a systematic review and meta-analysis of randomized controlled trials**

Pantaree Aswanetmanee, MD, Chok Limsuwat, MD, Kittipong Maneechotesuwan, MD,

Phunsup Wongsurakiat, MD

**Table S1** GRADE Summary of evidence

|  | | | | | | Summary of findings | | |  | |
| --- | --- | --- | --- | --- | --- | --- | --- | --- | --- | --- |
| Quality assessment | | | | | | No. of patients | | Effect | Strength | |
| Tracheal intubation rate | | | | | | | | | | |
| No. of studies | Risk of bias | Inconsistency | Indirectness | Imprecision | Publication  bias | NIV | COT/HFNC | RR^a^ (95% CI) | | **Low** |
| Seventeen RCTs | Serious^b^ | Serious^c^ | Not serious | serious | Possible^d^ | 288/930 | 354/871 | 0.68 (0.52,0.89) | |  |

Abbreviations: NIV, noninvasive ventilation; COT, conventional oxygen therapy; HFNC, high-flow nasal cannula; RR, risk ratio; CI, confidence interval. ^a^All data based on random effect models. ^b^No trials were blinded so all were deemed at high risk of bias. ^c^Moderate to high heterogeneity; I^2^ = 72.4%, P = 0.000. ^d^Possible existence of publication bias.

**Figure S1** Subgroup analysis according to immune status (nonimmunocompromised or immunocompromised patients): **i**ntubation rate in acute hypoxemic respiratory failure patients randomized to noninvasive ventilation (NIV) versus conventional oxygen therapy/high-flow nasal cannula (HFNC). Boxes and horizontal lines represent point estimates and 95% confidence intervals, varying in size according to the weight in the analysis

**Figure S2** Sensitivity analysis performed by removing 3 RCTs of which including some hypercapnic respiratory failure population: **i**ntubation rate in acute hypoxemic respiratory failure patients randomized to noninvasive ventilation (NIV) versus conventional oxygen therapy/high-flow nasal cannula (HFNC). Boxes and horizontal lines represent point estimates and 95% confidence intervals, varying in size according to the weight in the analysis

**Egger’s test: P = 0.032**

**Figure S3** Funnel plots for tracheal intubation rate in comparison between NIV and conventional oxygen therapy/high-flow nasal cannula (HFNC) groups
